# An SEIR model of influenza A virus infection and reinfection within a farrow-to-finish swine farm

**DOI:** 10.1371/journal.pone.0202493

**Published:** 2018-09-24

**Authors:** Fatima Etbaigha, Allan R. Willms, Zvonimir Poljak

**Affiliations:** 1 Department of Mathematics and Statistics, University of Guelph, Guelph, Ontario N1G 2W1 Canada; 2 Department of Population Medicine, University of Guelph, Guelph, Ontario N1G 2W1 Canada; University of Minnesota, UNITED STATES

## Abstract

Influenza A virus (IAV) in swine is a pathogen that causes a threat to the health as well as to the production of swine. Moreover, swine can spread this virus to other species including humans. The virus persists in different types of swine farms as evident in a number of studies. The core objectives of this study are (i) to analyze the dynamics of influenza infection of a farrow-to-finish swine farm, (ii) to explore the reinfection at the farm level, and finally (iii) to examine the effectiveness of two control strategies: vaccination and reduction of indirect contact. The analyses are conducted using a deterministic Susceptible-Exposed-Infectious-Recovered (SEIR) model. Simulation results show that the disease is maintained in gilts and piglets because of new susceptible pigs entering the population on a weekly basis. A sensitivity analysis shows that the results are not sensitive to variation in the parameters. The results of the reinfection simulation indicate that the virus persists in the entire farm. The control strategies studied in this work are not successful in eliminating the virus within the farm.

## Introduction

In 1918, the swine influenza A virus was recognized clinically in the United States which coincided with human influenza that caused about 20 million deaths around the world [1]. This zoonotic disease continues to be a public health concern due to the ability of the virus to spread readily and evolve [2]. Swine herds, which are recognized as reservoirs of IAV, can contribute to disease outbreak in other species [3]. This virus causes a respiratory disease in pigs with clinical signs including lethargy, coughing and nasal discharge [4, 5]. Currently, IAV has become endemic in the swine population around the world [2, 6–8], and it causes threats not only to the health but also to the production of swine [9].

Several factors affect the transmission of influenza virus in pigs including age, vaccination, and immunity levels [10]. Vaccination has often been used to minimize the spreading of IAV in pigs [11, 12]. However, it is still not clear whether vaccination is an effective strategy to reduce the virus in an entire swine herd [12, 13]. Additional studies have shown that maternally derived immunity can not only reduce clinical symptoms but also can be beneficial to reduce the spread of IAV in pigs. However, maternally derived immunity is effective only for a limited period of time [5, 14]. In spite of this research into IAV transmission, it is still not well understood how the dynamics of transmission operates at the level of pig population [13].

Many modelling approaches have been carried out to improve the understanding of disease dynamics in swine for infectious diseases, such as Salmonella [15], Pseudorabies [16] and Nipah virus [17]. In the context of influenza, although IAV has been frequently recorded in swine herds with risks to other animals and public health, there are still gaps in the information regarding the evaluation of IAV, and limited modelling studies conducted on IAV at the pig farm level [18].

Recently, a few articles on mathematical models of IAV spread in swine herds have been published. Pitzer et al. [19], developed a stochastic model of IAV in swine that showed a relation between the finishing herd size and seroprevalence but not between farrow-to-finish farm herd size and seroprevalence. They also examined the persistence of IAV in differently sized farms. Their findings indicated that as long as there is an inflow of new susceptible pigs to the farm, the virus persists even in small populations. Another stochastic approach of IAV in swine has been proposed by Cador et al. [20]. This work focuses on the effect of maternally derived immunity on IAV persistence in a farrow-to-finish farm. The results indicated that IAV in piglets can last a long time if maternal immunity is present. Additionally, Reynolds et al. [12], created a deterministic model to address the dynamics of IAV and the vaccination efficacy in USA breeding and wean-to-finish farms. Results showed that the disease is maintained in the breeding farm, while it becomes extinct in the wean-to-finish farm. Furthermore, the most common vaccination strategies did not prevent the spread of infection across the breeding farm. More recently, White et al. [21] proposed a stochastic model of IAV in a standard USA breeding farm. The authors tested different vaccination and management strategies and confirmed the finding of the persistence of IAV in the piglets population.

A study conducted by Poljack et al. [8, 22] confirmed that the influenza virus infection level is growing over the years in pig farms in Ontario. In this work we extend the deterministic SEIR model presented in [12] to suit the features of a standard Ontario commercial farrow-to-finish swine farm. This extension allow us to address the infection dynamics issue of IAV in this farm. In particular, our goals are to use this model to explore the persistence of the influenza virus, evaluate the reinfection at the farm level, and examine the effectiveness of vaccination and reduction of indirect contact at reducing the influenza virus infection through the farm. The model is structured to include the weekly progress of all pig growth stages including gilts, breeding sows, farrowing sows, and growing pigs. The assumptions of direct and indirect transmission between these different stages are considered in the model.

## Materials and methods

### Population and process

**Fig 1:**
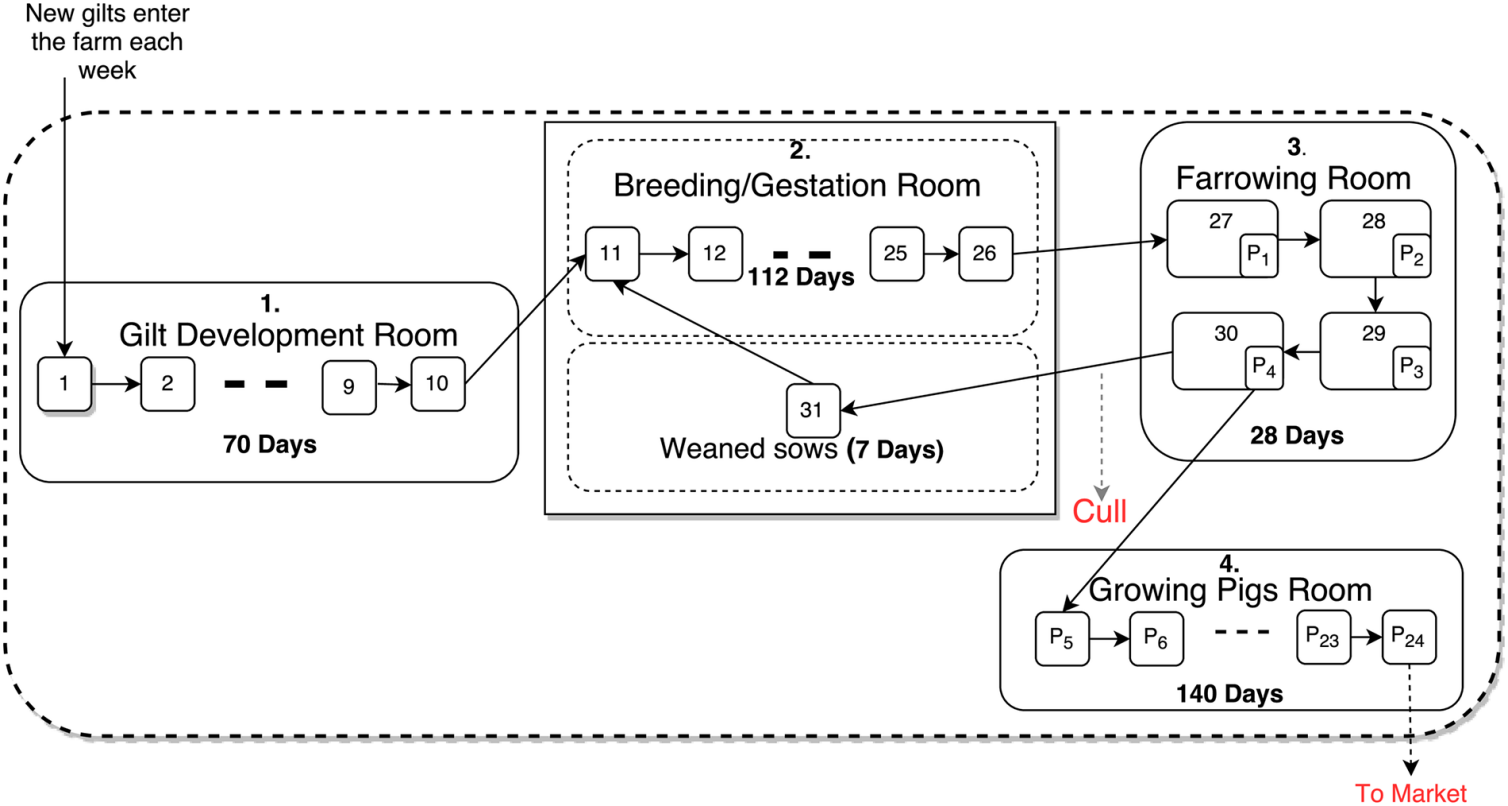
Standard commercial farrow-to-finish swine farm. This farm includes gilts, sows, piglets and growing pigs. They are housed in four buildings. In building 1, gilts enter the development room then they will go through building 2 (breeding/gestation room). Here, artificial insemination has been used for breeding. Then the pregnant sows will enter building 3 for farrowing (where they give birth through the first week). After culling, the weaned sows return to the breeding room where the cycle starts again. The piglets stay 4 weeks in building 3. After that, weaned piglets will move to the growing pigs room, then they leave the farm after 20 weeks.

As illustrated in Fig 1, the farrow-to-finish farm involves four types of animals: gilts (females that have not given birth yet), sows (females that have reproduced), piglets (young pigs less than 4 weeks old) and growing pigs (pigs from weaning to marketing level). The production in the farm uses a system of rooms that are associated with four stages of a pig’s life cycle: gilt development stage, breeding/gestation stage, farrowing stage and growing stage [23]. The pigs in each of these stages are divided into (weekly) age classes, where the pigs in the last age class of each stage enter the first age class of the next stage as shown in Fig 1. Furthermore, pigs of different ages or reproductive status can be grouped into one room, for example the farrowing room contains sows and piglets and the breeding/gestation room contains weaned and pregnant sows.

New gilts enter the gilt development room each week and remain there for 10 weeks (70 days) until they join the sows in the breeding/gestation room. At that time, they are artificially inseminated. The reproductive cycle of sows is 147 days. The pregnant sows spend 112 days in the breeding/gestation room, then they are moved to the farrowing room a few days before the expected day of birthing. In the farrowing room, sows nurse their piglets for 4 weeks (28 days) until weaning. Then the individual weaned sows are either culled or moved back to the breeding room, where they stay 7 days until insemination and then they start a new cycle again.

Note that the pregnancy period of sows is typically 115 days [24]. In our model, sows get pregnant at the beginning of week class 11, plus or minus few days. The pregnant sows spend week classes 11 to 26 in the breeding/gestation room (112 days), then they move to the farrowing room and give birth in days 2 to 7 of week class 27.

The sows give birth to an average of 12 piglets per sow. Once the piglets are weaned in piglets week class 4 (*P*_4_), they are moved directly to the growing room for 140 days, at which point they are transported for slaughter.

### Construction of the model

The infection and reinfection process of IAV in the farrow-to-finish farm is represented by an SEIR model. The SEIR model presented in [12] is extended to include the group of growing pigs. This is necessary since the previously proposed model in [12] studied a breeding farm which does not include the growing pigs group.

In this model, the compartments are selected based on the disease characteristics and age status. For gilts and sows, *S*_*i*_(*t*), *E*_*i*_(*t*), *I*_*i*_(*t*) and *R*_*i*_(*t*) are the number of susceptible, exposed, infectious, and recovered, respectively; *t* is the time, which is measured in days, where *t* ≥ 0, and *i* represents the week class. For the piglets and growing pigs, we specify the week class with *j*, and add a superscripts *p*: $S_{j}^{p}\left(t\right)$, $E_{j}^{p}\left(t\right)$, $I_{j}^{p}\left(t\right)$ and $R_{j}^{p}\left(t\right)$. Furthermore, for piglets and growing pigs with immunity, the superscripts are changed to *pm*: $S_{j}^{pm}\left(t\right)$, $E_{j}^{pm}\left(t\right)$, $I_{j}^{pm}\left(t\right)$ and $R_{j}^{pm}\left(t\right)$. All the individuals within the farm move from the susceptible pigs population to exposed pigs population due to either direct contact or indirect contact. The direct contact comes from the infectious pigs in the same room, while indirect contact comes from infectious pigs in other rooms of the farm. The individuals in the exposed class move to the infectious class at some latency rate *σ*. After entering the infectious class, the individuals recover at some recovery rate *γ*. Lastly, the individuals who have recovered, return back to susceptible class at some immunity rate *ω*. Pigs enter the farm as cohort into class 1. Each week that cohort move to subsequent number class. The sows after give birth move back to class 11. piglets enter class 5 in growing room where they have different immunity. Each location in the farm has different classes of pigs (see Table 1). This table extends the corresponding table in [12].

**Table 1:** Class of pigs corresponding to each location.

Class	Population variables	Animal	Location
*i* ∈ {1, 2, …10}	*S*_*i*_, *E*_*i*_, *I*_*i*_, *R*_*i*_	Gilts	Gilt development room
*i* ∈ {11, 12, …26}	−	Pregnant sows	Breeding/gestation room
*i* ∈ {27, 28, 29, 30}	−	Farrowing sows	Farrowing room
*i* ∈ {31}	−	Weaned sows	Breeding/gestation room
*j* ∈ {1, 2, 3, 4}	$S_{j}^{p},E_{j}^{p},I_{j}^{p},R_{j}^{p}$	Piglets	Farrowing room
*j* ∈ {5, 6, …, 24}	$S_{j}^{p},E_{j}^{p},I_{j}^{p},R_{j}^{p}$	Growing pigs	Growing pigs room

Here we describe the model of the reinfection within the farrow-to-finish swine farm. In order to model the reinfection scenario, similar to [12], we assume that the recovered animals can become susceptible to infection again at an average duration of immunity 1/*ω* days. To evaluate this scenario, the parameter *ω* is introduced into the equations to represent the average rate of the immunity waning after the first infection.

Formally, the SEIR model of sows and gilts are given by the following ordinary differential equations (ODEs) system:

$$\frac{dS_{i}}{dt}=-\beta_{d}I_{d_{i}}S_{i}-\beta_{ind_{1}}I_{ind_{i}}S_{i}-\beta_{ind_{2}}I_{ind}^{*}S_{i}-\mu S_{i}+\omega R_{i},$$ (1)

$$\frac{dE_{i}}{dt}=\beta_{d}I_{d_{i}}S_{i}+\beta_{ind_{1}}I_{ind_{i}}S_{i}+\beta_{ind_{2}}I_{ind}^{*}S_{i}-\left(\mu +\sigma \right)E_{i},$$ (2)

$$\frac{dI_{i}}{dt}=\sigma E_{i}-\left(\mu +\gamma \right)I_{i},$$ (3)

$$\frac{dR_{i}}{dt}=\gamma I_{i}-\left(\mu +\omega \right)R_{i},$$ (4)

where *i* ∈ {1, 2, ….10} for gilts and *i* ∈ {11, 12, …., 31} for sows. Furthermore,

$$I_{d_{i}}=\sum_{k=1}^{10}I_{k}\;\forall \;i\in \left\{1,2,....10\right\},$$ (5)

$$I_{d_{i}}=\sum_{k=11}^{26}I_{k}+I_{31}\;\forall \;i\in \left\{11,12,....26,31\right\},$$ (6)

$$I_{d_{i}}=\sum_{k=27}^{30}I_{k}+\sum_{j=1}^{4}I_{j}^{p}\;\forall \;i\in \left\{27,28,29,30\right\},$$ (7)

$$I_{ind_{i}}=\sum_{k=11}^{31}I_{k}+\sum_{j=1}^{4}I_{j}^{p}\;\forall \;i\in \left\{1,2,....10\right\},$$ (8)

$$I_{ind_{i}}=\sum_{k=1}^{10}I_{k}+\sum_{k=27}^{30}I_{k}+\sum_{j=1}^{4}I_{j}^{p}\;\forall \;i\in \left\{11,12,....26,31\right\},$$ (9)

$$I_{ind_{i}}=\sum_{k=1}^{26}I_{k}+I_{31}\;\forall \;i\in \left\{27,28,29,30\right\},$$ (10)

$$I_{ind}^{*}=\sum_{j=5}^{24}I_{j}^{p}\;\text{for}\;\text{all}\;\text{class}\;\text{of}\;\text{gilts}\;\text{and}\;\text{sows}.$$ (11)

The direct and indirect transmission rates are defined respectively as the parameters *β*_*d*_, $\beta_{ind_{1}}$ and $\beta_{ind_{2}}$. All governing parameters are stated in Table 2.

**Table 2:** Parameters stated in the IAV infection model with descriptions and values. Parameter values are taken from [12], except for *ω* which is taken from [20], and $\beta_{ind_{2}}$, $\beta_{ind_{2}}^{pm}$, $\beta_{ind_{2}}^{p}$ and $\beta_{ind_{2}}^{gm}$ which are an assumption.

Description	Parameter	Value (Range)
Direct transmission rate for gilts, sows and growing pigs	*β*_*d*_	0.285 (0.091 − 0.9) *day*^−1^
Indirect transmission rate for gilts and sows	$\beta_{ind_{1}}$	0.0016 *day*^−1^ = (*β*_*d*_/178)
Indirect transmission rate for gilts, sows and growing pigs	$\beta_{ind_{2}}$	0.00057 *day*^−1^ = (*β*_*d*_/500)
Natural death rate of sows and gilts	*μ*	0.0004 *day*^−1^
Direct transmission rate for piglets	$\beta_{d}^{p}$	0.218 (0.147 − 0.310) *day*^−1^
Indirect transmission rate for piglets	$\beta_{ind_{1}}^{p}$	0.001 *day*^−1^ = $\left(\beta_{d}^{p}/178\right)$
Direct transmission rate for piglets with maternal immunity	$\beta_{d}^{pm}$	0.014 (0.001 − 0.061) *day*^−1^
Indirect transmission rate for piglets with maternal immunity	$\beta_{ind_{1}}^{pm}$	0.00008 *day*^−1^ = $\left(\beta_{d}^{pm}/178\right)$
Indirect transmission rate for piglets	$\beta_{ind_{2}}^{p}$	0.00044 *day*^−1^ = $\left(\beta_{d}^{p}/500\right)$
Indirect transmission rate for piglets with maternal immunity	$\beta_{ind_{2}}^{pm}$	0.000028 *day*^−1^ = $\left(\beta_{d}^{pm}/500\right)$
Natural death rate for piglets	*μ*^*p*^	0.005 *day*^−1^
Average of latent period	1/*σ*	*σ* = 1/2 *day*^−1^
Average of infectious period	1/*γ*	*γ* = 1/5 *day*^−1^
Birth rate	*b*	12 births per litter per sow
Immunity waning after the first infection	1/*ω*	*ω* = 1/180 *day*^−1^
Direct transmission rate for growing pigs with maternal immunity (depends on time)	$\beta_{d}^{gm}=\beta^{gm(T)}$	*β*_*d*_(1.01 − 0.96 *e*^−0.06*T*^)*day*^−1^
Indirect transmission rate for growing pigs with maternal immunity	$\beta_{ind_{2}}^{gm}$	$\left(\beta_{d}^{gm}/500\right)$
Natural death rate for growing pigs	*μ*^*g*^	0.00028 *day*^−1^

For piglets, we divide the piglets into two groups: one inherits the maternal immunity and the other does not as in [12]. For case without maternal immunity, we assume that only the susceptible, exposed and infectious sows in week class 27 give birth, while in the case with maternal immunity, we assume that only the recovered sows give birth. The equations for piglets without maternal immunity are as follows:

$$\begin{array}{cc} \frac{dS_{j}^{p}}{dt}= & b_{j}\left(S_{27}+E_{27}+I_{27}\right)-\beta_{d}^{p}I_{d}S_{j}^{p}\\ & -\beta_{ind_{1}}^{p}I_{ind}S_{j}^{p}-\beta_{ind_{2}}^{p}I_{ind}^{*}S_{j}^{p}-\mu^{p}S_{j}^{p}+\omega R_{j}^{p}+\omega R_{j}^{pm}, \end{array}$$ (12)

$$\frac{dE_{j}^{p}}{dt}=\beta_{d}^{p}I_{d}S_{j}^{p}+\beta_{ind_{1}}^{p}I_{ind}S_{j}^{p}+\beta_{ind_{2}}^{p}I_{ind}^{*}S_{j}^{p}-\left(\mu^{p}+\sigma \right)E_{j}^{p},$$ (13)

$$\frac{dI_{j}^{p}}{dt}=\sigma E_{j}^{p}-\left(\mu^{p}+\gamma \right)I_{j}^{p},$$ (14)

$$\frac{dR_{j}^{p}}{dt}=\gamma I_{j}^{p}-\left(\mu^{p}+\omega \right)R_{j}^{p}.$$ (15)

For piglets with maternal immunity group are:

$$\begin{array}{cc} \frac{dS_{j}^{pm}}{dt}= & b_{j}\left(R_{27}\right)-\beta_{d}^{pm}I_{d}S_{j}^{pm}-\beta_{ind_{1}}^{pm}I_{ind}S_{j}^{pm}\\ & -\beta_{ind_{2}}^{pm}I_{ind}^{*}S_{j}^{pm}-\mu^{p}S_{j}^{pm}, \end{array}$$ (16)

$$\frac{dE_{j}^{pm}}{dt}=\beta_{d}^{pm}I_{d}S_{j}^{pm}+\beta_{ind_{1}}^{pm}I_{ind}S_{j}^{pm}+\beta_{ind_{2}}^{pm}I_{ind}^{*}S_{j}^{pm}-\left(\mu^{p}+\sigma \right)E_{j}^{pm},$$ (17)

$$\frac{dI_{j}^{pm}}{dt}=\sigma E_{j}^{pm}-\left(\mu^{p}+\gamma \right)I_{j}^{pm},$$ (18)

$$\frac{dR_{j}^{pm}}{dt}=\gamma I_{j}^{pm}-\left(\mu^{p}+\omega \right)R_{j}^{pm},$$ (19)

where *j* ∈ {1, 2, 3, 4}, for both cases. Furthermore,

$$I_{d}=\sum_{i=27}^{30}I_{i}+\sum_{j=1}^{4}I_{j}^{p},$$ (20)

$$I_{ind}^{*}=\sum_{j=5}^{24}I_{j}^{p},$$ (21)

$$I_{ind}=\sum_{i=1}^{26}I_{i}+I_{31}.$$ (22)

*b*_*j*_ is the birth rate. Since weekly average birth number of sows is 12 piglets between days 2 and 7, therefore,

$$\;b_{1}\left(t\right)=\{\begin{array}{c} 0\;\;\;\text{if}\;\;0\leq mod(t,7)<2\\ b/5\;\;\text{if}\;\;2\leq mod(t,7)\leq 7, \end{array}$$

where *b*_*j*_ = 0 ∀ *j* ∈ {2, 3, 4}.

Eqs (16)–(19) represent classes of piglets who have inherited immunity from their mother. In the case of the with immunity group, the direct transmission rate $\beta_{d}^{pm}$ is assumed to be the same for all piglets since the material immunity started to decay at age 3 weeks [25]. See Table 2 for the description of the parameters that are involved in these equations.

Corresponding to this case, the growing pigs are also separated into two groups: one with maternal immunity and another without. The equations for pigs with maternal immunity are:

$$\frac{dS_{j}^{pm}}{dt}=-\beta_{d}^{gm}I_{d}^{p}S_{j}^{pm}-\beta_{ind_{2}}^{gm}I_{ind}^{p}S_{j}^{pm}-\mu^{g}S_{j}^{pm},$$ (23)

$$\frac{dE_{j}^{pm}}{dt}=\beta_{d}^{gm}I_{d}^{p}S_{j}^{pm}+\beta_{ind_{2}}^{gm}I_{ind}^{p}S_{j}^{pm}-\left(\mu^{g}+\sigma \right)E_{j}^{pm},$$ (24)

$$\frac{dI_{j}^{pm}}{dt}=\sigma E_{j}^{pm}-\left(\mu^{g}+\gamma \right)I_{j}^{pm},$$ (25)

$$\frac{dR_{j}^{pm}}{dt}=\gamma I_{j}^{pm}-\left(\mu^{g}+\omega \right)R_{j}^{pm},$$ (26)

where *j* ∈ {5, …, 14}. Furthermore,

$$I_{d}^{p}=\sum_{j=5}^{24}I_{j}^{p},$$ (27)

$$I_{ind}^{p}=\sum_{i=1}^{31}I_{i}+\sum_{j=1}^{4}I_{j}^{p}.$$ (28)

The weaned piglets with maternal immunity will enter these classes at age approximately 21 days when the maternal immunity starts to wane. Furthermore, the maternal antibodies will decay to zero by age 13 weeks old [25]. Therefore to represent this waning, we consider the direct transmission rate for these ten classes of pigs is depending on time i.e. $\beta_{d}^{gm}=\beta^{gm}\left(T\right)$, where *T* = age of pig −21 days, and we set $\beta_{ind_{2}}^{gm}=\beta_{d}^{gm}/500$ (see Table 2).

The equations of growing pigs without maternal immunity are:

$$\frac{dS_{j}^{p}}{dt}=-\beta_{d}I_{d}^{p}S_{j}^{p}-\beta_{ind_{2}}I_{ind}^{p}S_{j}^{p}-\mu^{g}S_{j}^{p}+\omega R_{j}^{p}+\omega R_{j}^{pm},$$ (29)

$$\frac{dE_{j}^{p}}{dt}=\beta_{d}I_{d}^{p}S_{j}^{p}+\beta_{ind_{2}}I_{ind}^{p}S_{j}^{p}-\left(\mu^{g}+\sigma \right)E_{j}^{p},$$ (30)

$$\frac{dI_{j}^{p}}{dt}=\sigma E_{j}^{p}-\left(\mu^{g}+\gamma \right)I_{j}^{p},$$ (31)

$$\frac{dR_{j}^{p}}{dt}=\gamma I_{j}^{p}-\left(\mu^{g}+\omega \right)R_{j}^{p},$$ (32)

where *j* ∈ {5, …, 14}.

The piglets without immunity will enter these classes for *j* ∈ {5, …, 14}. Then both groups with immunity(after losing their immunity) and without will move to next classes *j* ∈ {15, ‥, 24} with the same equations as (29)–(32) to spend another 10 weeks in the growing room where we consider $R_{j}^{pm}=0$.

Note that for the no reinfection case, we set all *ω* = 0 in the above equations. All these equations are solved together using the ODE45 solver on weekly basis, thus the time span of the solver is set to 0 ≤ *t* ≤ 7. The solutions provided at the end of the time span for each week represent the number of pigs in every particular class. These solutions are then used to apply the farm dynamics. As illustrated in Fig 1, pigs in class *i* move to class *i* + 1, 1 ≤ *i* ≤ 30 and pigs in class 31 move to class 11. Pigs in class *j* move to class *j* + 1, 1 ≤ *i* ≤ 23, and pigs in class *j* = 24 leave the farm. Once all movements of the pigs are completed, the current status of the farm represent the initial conditions for the next week. This cycle is repeated for the desired number of weeks.

### Disease management practices

A goal of this paper is to examine the effectiveness of the two control strategies: vaccination, and reduction of indirect contact, by identifying whether these two strategies help in reducing the virus within the farm.

To model the vaccination strategies, the susceptible animals only are moved to a recovered state where reinfection can occur. We test effectiveness of the vaccination when the disease is endemic in the farm, i.e. at some time after the system reaches the equilibrium. We assume that the effect of vaccine wanes at the same rate as natural immunity. We test the effectiveness of four common ways of vaccination: 1) vaccinating only the incoming gilts each week, 2) pre-farrow vaccination of the pregnant sows each weak so that the piglets will obtain passive maternal immunity through colostrum from their mother, 3) vaccinating the piglets at birth, and 4) mass vaccination, where all gilts, sows, piglets and growing pigs are vaccinated once at the same time.

To reduce indirect contact, the farm can apply various preventive measures such as reducing the movement of people and equipment between rooms, and cleaning the boots and clothes of the farm workers regularly. These measures can help prevent disease transmission between the rooms. In the model, this scenario is achieved by reducing *β*_*ind*_ in the whole farm to the best possible reduction case which is equal to zero.

### Model parameters and farm assumptions

All model parameters are stated in Table 2. The model parameters and their values presented in [12] are used here except *ω* which is taken from [20]. $\beta_{ind_{2}}$, $\beta_{ind_{2}}^{pm}$, $\beta_{ind_{2}}^{p}$ and $\beta_{ind_{2}}^{gm}$ are new parameters in the SEIR model. In this farm, the room for growing pigs is likely a greater distance away from the rest of the rooms than the other rooms are from each other. For this purpose, we assume the values of these new transmission rates are scaled by 500. (e.g., $\beta_{ind_{2}}=\beta_{d}/500$). Scaling these rates by 500 will allow more effective contact between the different age groups than the ones reported by Evans et al. [26] in which they scale these values by 10^−3^ and 10^−4^.

Regarding the farm population and farm dynamics, in this study, we assume the farm contains 646 sows and 50 gilts. This farm size is about the same size as average farm size in Ontario [23]. In addition, farm with this number of sows in a sow herd is more likely to have animals of all age groups on the same premises (i.e site or geographical location). For this sow herd with 100% farrowing rate and 2.48 annual litters per sow (based on 365 days/147 days of reproductive cycle), the weekly starting number of sows that enter the breeding room is 31 (based on total number of sows * litters per sow per year/farrowing rate/52 weeks). Additionally, we assume that the annual rate of replacement of sows in the farm is 40%. Then the number of gilts to be introduced weekly in the farm is 5 (based on the weekly starting number of sows * sow replacement rate/ litters per sow per year). We also assume that the population size is constant, therefore it is assumed that the number of culled sows plus the natural death each week is 5, which is equal to the number of gilts introduced weekly. These calculations are based on [23]. The weekly starting number of sows (31) will yield approximately 359 piglets each week. Therefore, the total number of piglets in the farm is approximately 1039 to 1361, while the total number of growing pigs is approximately 6640.

The developed SEIR model has been numerically simulated on a farrow-to-finish system. As described above, the movement of the pigs is on a weekly basis. The farm has been initialized as a fully populated farm with all individuals in the susceptible state. For the sows, we set the susceptible initial conditions such that every class of them is initialized according to the natural death rate of the class age.

$$S_{i}\left(0\right)=S_{i-1}\left(0\right)\;.\;e^{-7\mu }\;\forall \;i\in \left\{12,13,....30\right\}.$$

For week class 11, the susceptible initial condition is

$$S_{11}\left(0\right)=S_{31}\left(0\right)\;.\;e^{-7\mu }+S_{10}\left(0\right)\;.\;e^{-7\mu },$$

the susceptible initial condition for week class 31 is

$$S_{31}\left(0\right)=\left(S_{30}\left(0\right)\;.\;e^{-7\mu }\right)-\text{cull},$$

$$\text{cull}=(646\;.\;e^{-7\mu }+5\;.\;e^{-70\mu })-646.$$

$$E_{i}\left(0\right)=0,\;I_{i}\left(0\right)=0\;\text{and}\;R_{i}\left(0\right)=0\;\forall \;i\in \left\{11,12,....31\right\}.$$

For piglets with immunity, we start with the initial conditions

$$(S_{j}^{pm}\left(0\right),E_{j}^{pm}\left(0\right),I_{j}^{pm}\left(0\right),R_{j}^{pm}\left(0\right))=\left(0,0,0,0\right)\;\text{for}\;\text{all}\;\text{classes}.$$

As for the piglets without immunity, we set the initial condition for *j* = 1 to

$$(S_{1}^{p}\left(0\right),E_{1}^{p}\left(0\right),I_{1}^{p}\left(0\right),R_{1}^{p}\left(0\right))=\left(0,0,0,0\right),$$

and the initial conditions for the rest can be formulated as follows:

$$S_{j}^{p}\left(0\right)=372\;.\;e^{-(\left(j-1\right)\;\times \;7\;\mu^{p})}\;\forall \;j\in \left\{2,3,4\right\},$$

$$E_{j}^{p}\left(0\right)=0,\;I_{j}^{p}\left(0\right)=0\;\text{and}\;R_{j}^{p}\left(0\right)=0\;\forall \;j\in \left\{2,3,4\right\}.$$

The initial conditions for the growing pigs are

$$S_{j}^{p}\left(0\right)=S_{j-1}^{p}\left(0\right).\;e^{-7\mu^{g}}\;\forall \;j\in \left\{\;5,6....24\right\},$$

$$E_{j}^{p}\left(0\right)=0,\;I_{j}^{p}\left(0\right)=0\;\text{and}\;R_{j}^{p}\left(0\right)=0\;\forall \;j\in \left\{\;5,6....24\right\}.$$

Finally the gilts are initialized with 5 susceptible for the first week class *S*_1_(0) = 5, and for next age classes, the initial conditions are

$$S_{i}\left(0\right)=S_{i-1}\left(0\right)\;.\;e^{-7\mu }\;\forall \;i\in \left\{2,3....,10\right\},$$

$$E_{i}\left(0\right)=0,\;I_{i}\left(0\right)=0\;\text{and}\;R_{i}\left(0\right)=0\;\forall \;i\in \left\{2,3....,10\right\}.$$

To start our simulation, we consider only one infected individual in the gilts entering week class 1, so the initial condition for week class one becomes

$$(S_{1}\left(0\right),E_{1}\left(0\right),I_{1}\left(0\right),R_{1}\left(0\right))=\left(4,0,1,0\right).$$

The ordinary differential equation solver has been used to solve the system of ODEs for our model (ode45 solver using MATLAB 2016). At the end of each week, movements of the pigs are implemented as described in Fig 1: five susceptible gilts enter the farm and the growing pigs at the final stage leave the farm.

### Sensitivity analysis

To evaluate the sensitivity of the model to the parameters, we vary the values of all direct and indirect transmission rates for the pigs in the farm. The main control transmission parameters are *β*_*d*_, $\beta_{d}^{p}$, and $\beta_{d}^{pm}$, and all the other transmission parameters are computed based on these control transmission parameters as shown in Table 2. For each control parameter, the values are varied as shown in Table 2. To evaluate the effect of each control parameter, we uniformly sample 100 different values from the control parameter range at equal interval. All the other parameters in the model such as *γ* and *σ* are fixed as they are determined by the disease and are not related to the farm structure or management strategy [12].

## Results

### Infection dynamics in the farm

**Fig 2:**
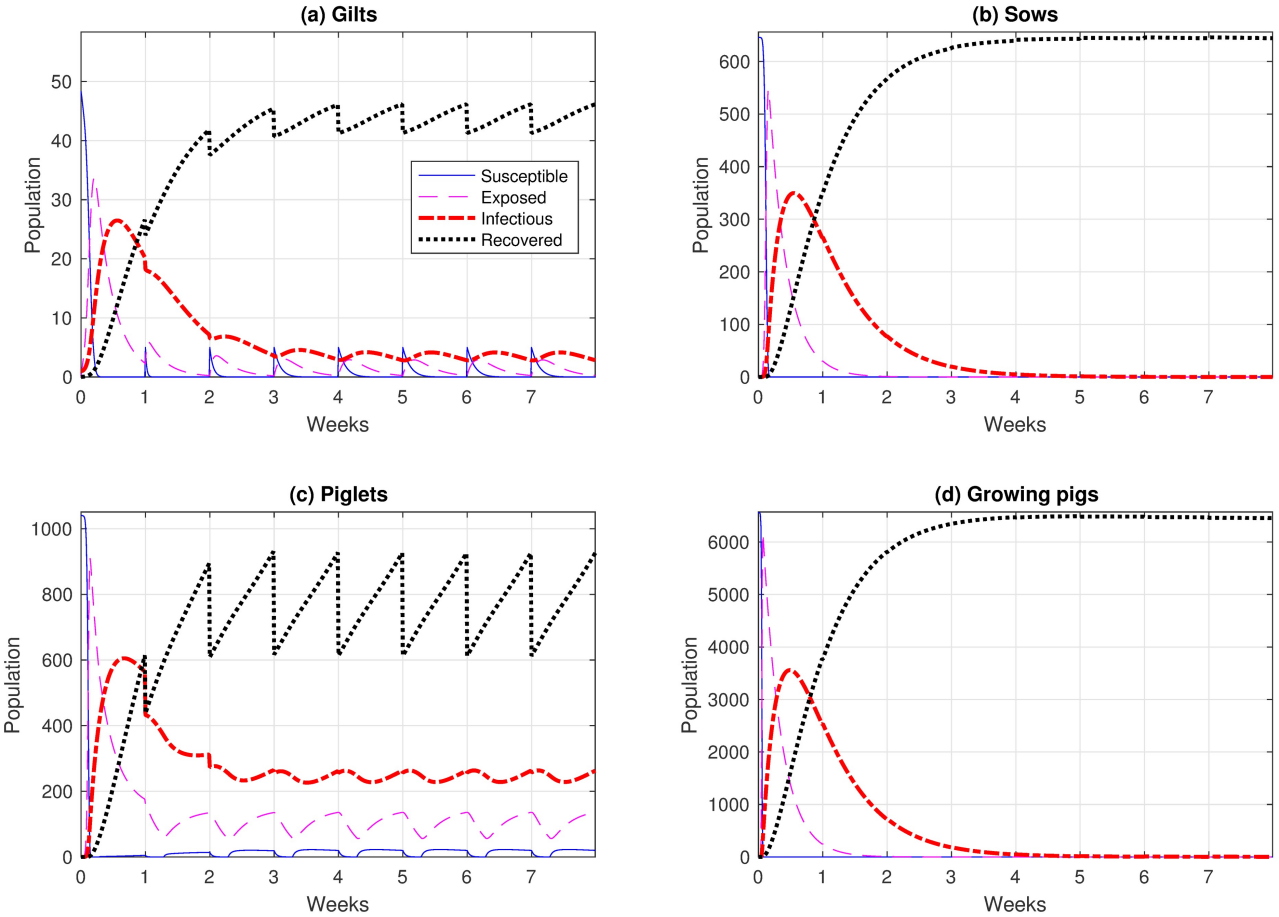
Influenza dynamics in a farrow-to-finish swine farm for (*a*) gilts, (*b*) sows, (*c*) piglets and (*d*) growing pigs. In panel (*a*) all 10 classes of gilts are combined into one group. In panel (*b*) all 21 classes of sows are combined into one group. In panel (*c*) all 4 classes of piglets are combined into one group. In panel (*d*) all 20 classes of growing pigs are combined into one group.

For the no reinfection case, we set *ω* = 0 in all our equations. Based in our model, we found that a single virus introduced to gilts spreads quickly in the farm as evident in Fig 2. For gilts, Fig 2a shows rapid reduction in the number of susceptible gilts until none of them is susceptible, at which time gilts change state to one of the other non-susceptible states. After a few days, about 50% of the gilts are infectious, after which a decline in the number of infectious gilts is observed, eventually resulting in all gilts recovering and never getting infectious again. The equilibrium is reached at week 3 with only 3 infectious gilts (approximately 6% of the gilts), and most of the rest of the gilt population having recovered. The oscillating behavior of this equilibrium is related to the introduction of new susceptible gilts every week. In contrast, Fig 2b shows that the number of infectious sows diminishes to zero by approximately 14 days after the peak.

For piglets, they become infectious immediately once the virus is spread in the farm (see Fig 2c). Then the number of infectious piglets starts to decline until it reaches the cycle equilibrium. The equilibrium is reached after approximately 4 weeks; at the equilibrium, approximately 25% of the piglets are infectious. The susceptible piglets are all from the piglets with immunity group. This is due to the fact that the piglets with immunity do not become infectious immediately.

For growing pigs, as evident in Fig 2d, the number of infectious animals decreases to zero after the initial peak. In our study, no difference is observed between the growing pigs with immunity and without immunity (not shown) since most of the incoming pigs had already recovered.

### Reinfection

**Fig 3:**
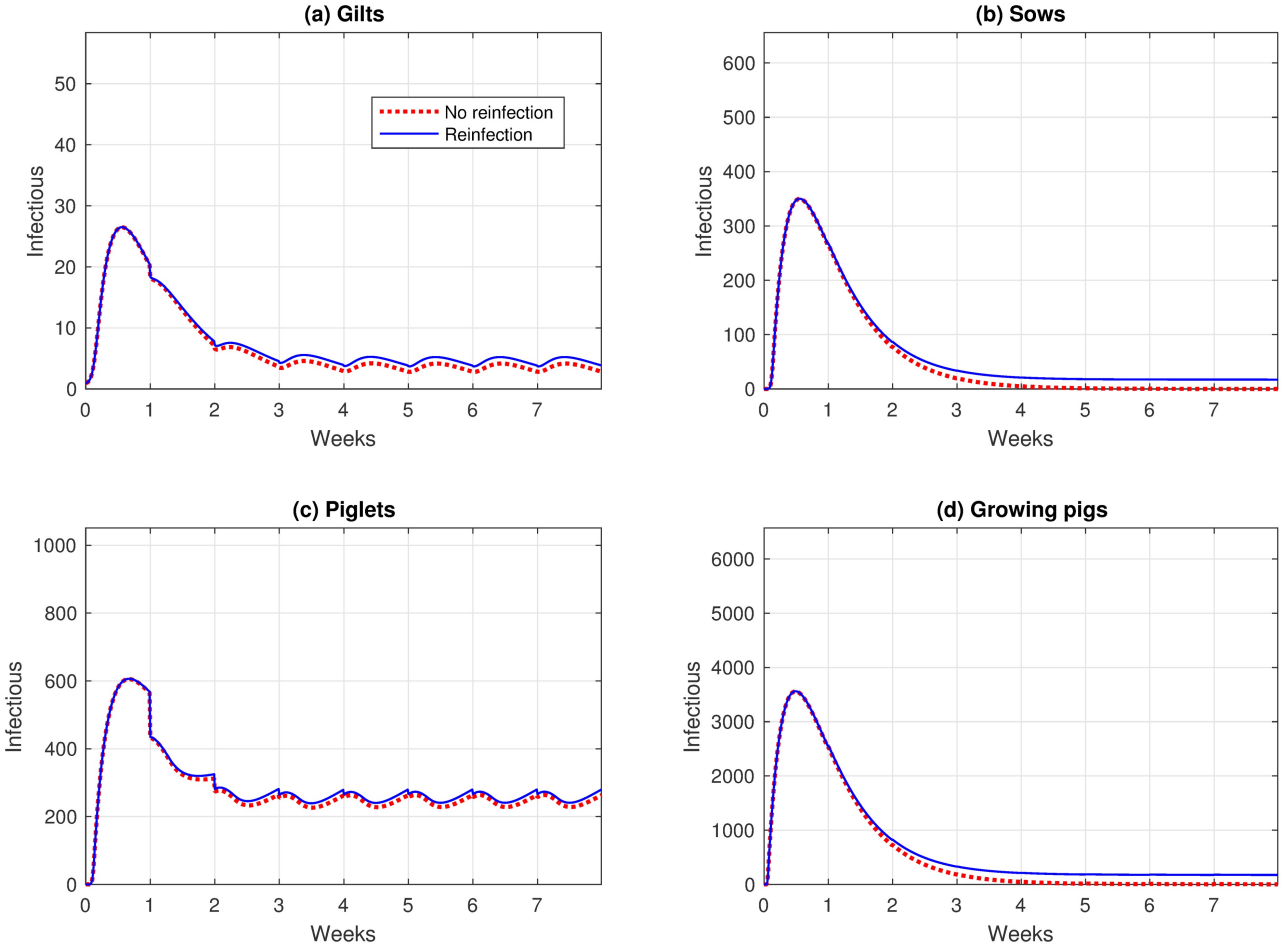
Infectious levels for (*a*) gilts, (*b*) sows, (*c*) piglets and (*d*) growing pigs under the no reinfection and reinfection scenarios.

In the reinfection scenario, our model reflects the condition where the individuals can re-enter the susceptible state once recovered. The animals in the susceptible state include some that have moved from the previous room and some that have moved from the recovered state back to the susceptible state. This movement from recovered to susceptible results in a slight increase in the number of infectious animals in the whole farm compared to that number in the no reinfection scenario (see Fig 3).

### Testing vaccination strategies

**Fig 4:**
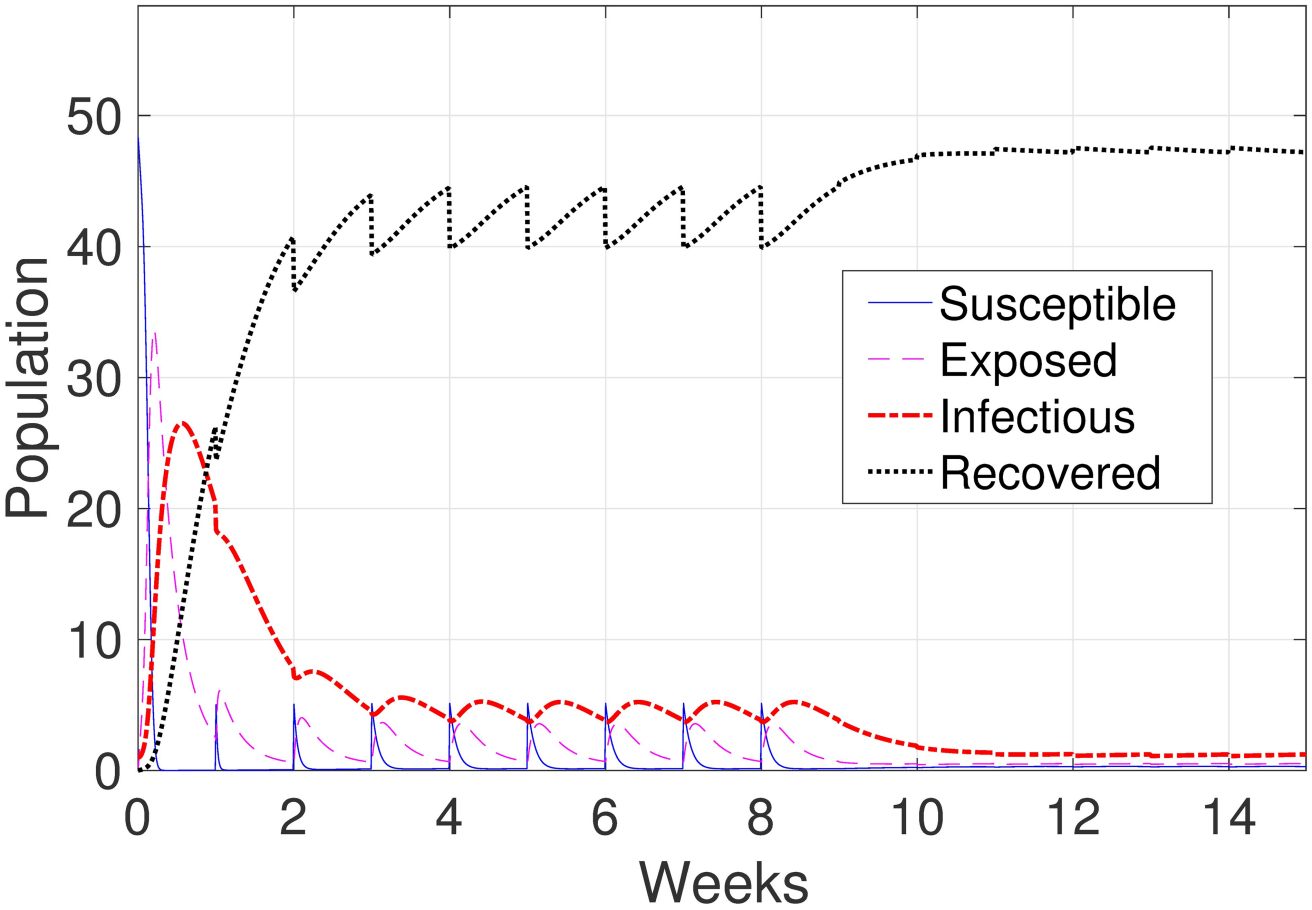
The effect of vaccinating incoming gilts each week starting at the end of week 8. The panel shows a reduction in the number of infectious gilts after the vaccination.

**Fig 5:**
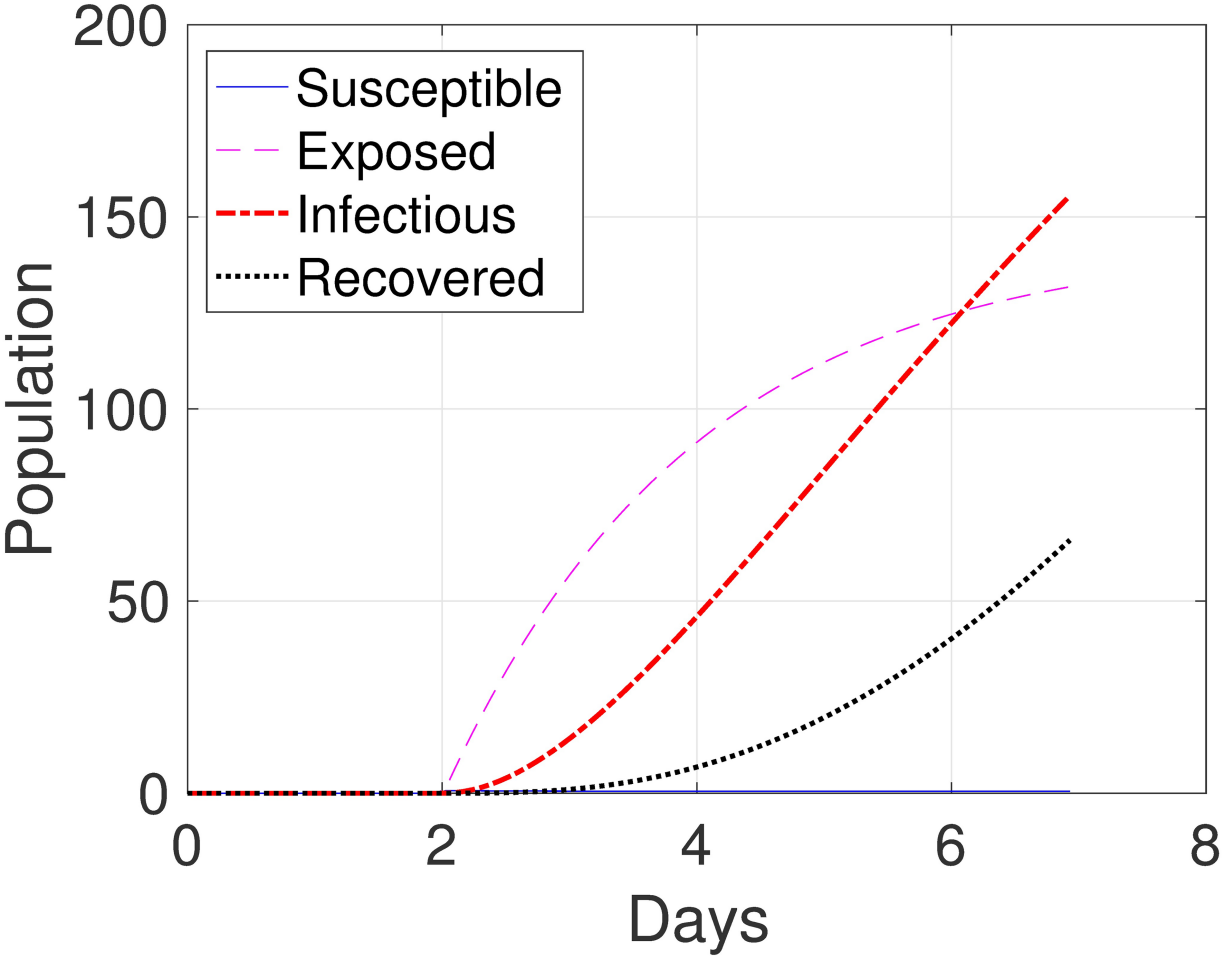
The panel shows the piglets at week class one where most of them are infectious once they are born in day two.

**Fig 6:**
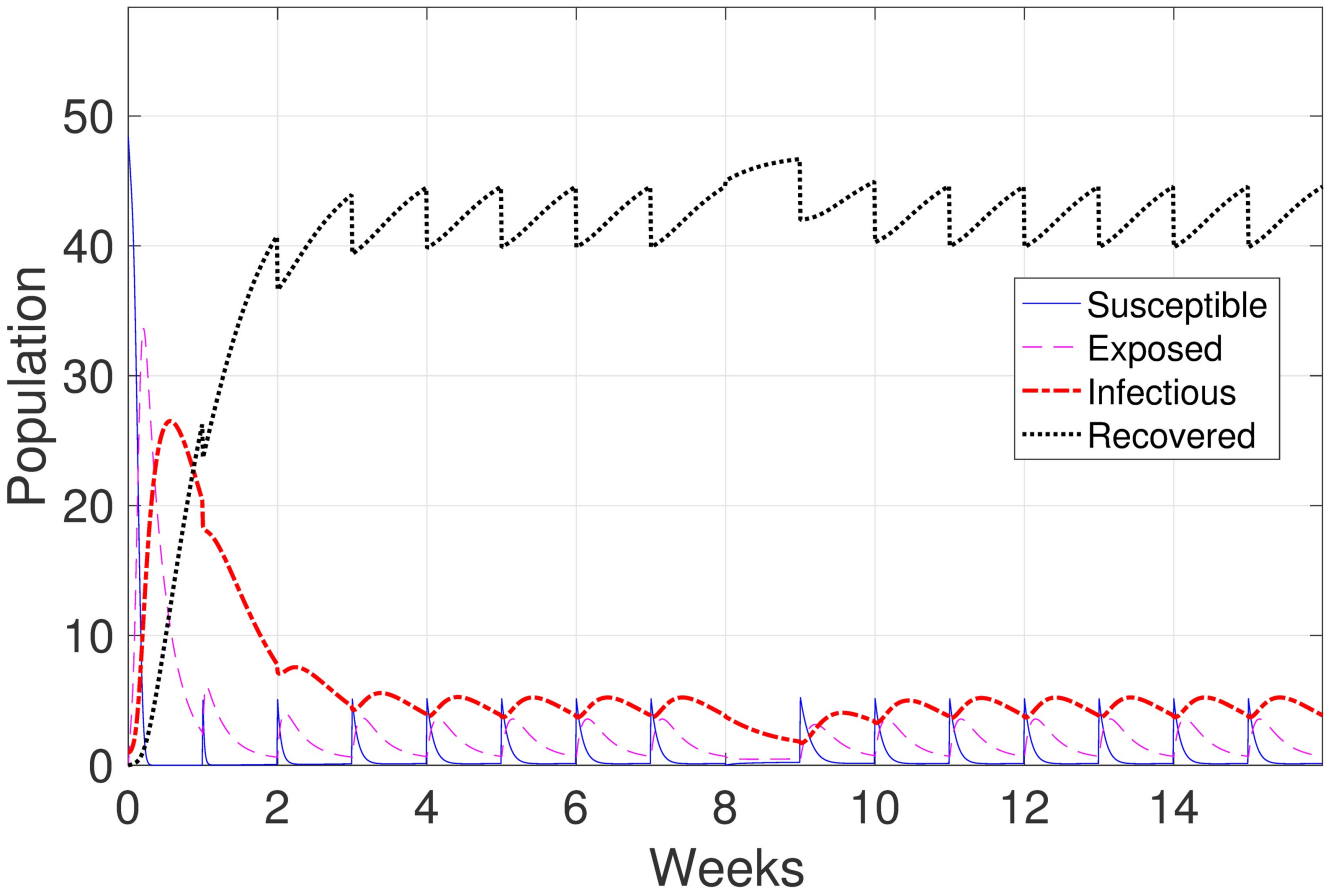
Gilts mass vaccination at week 8.

For testing the vaccination strategies, we model the case when the vaccination is administered after the disease is already present on the farm. In our model, vaccinating incoming gilts results in a reduction in the number of infectious gilts, as illustrated in Fig 4. Pre-farrow vaccination, as implemented in our model, does not show any change in the number of infectious animals in the piglets. Vaccinating the piglets at the end of week class 1 also shows no significant effect, as all the piglets without immunity become infectious as soon as they are born (see Fig 5). For the mass vaccination, only the number of infectious gilts is decreed during the vaccination week as can be seen in Fig 6, the gilts return to the regular endemic equilibrium after the vaccination week.

### Reduction of indirect contact

**Fig 7:**
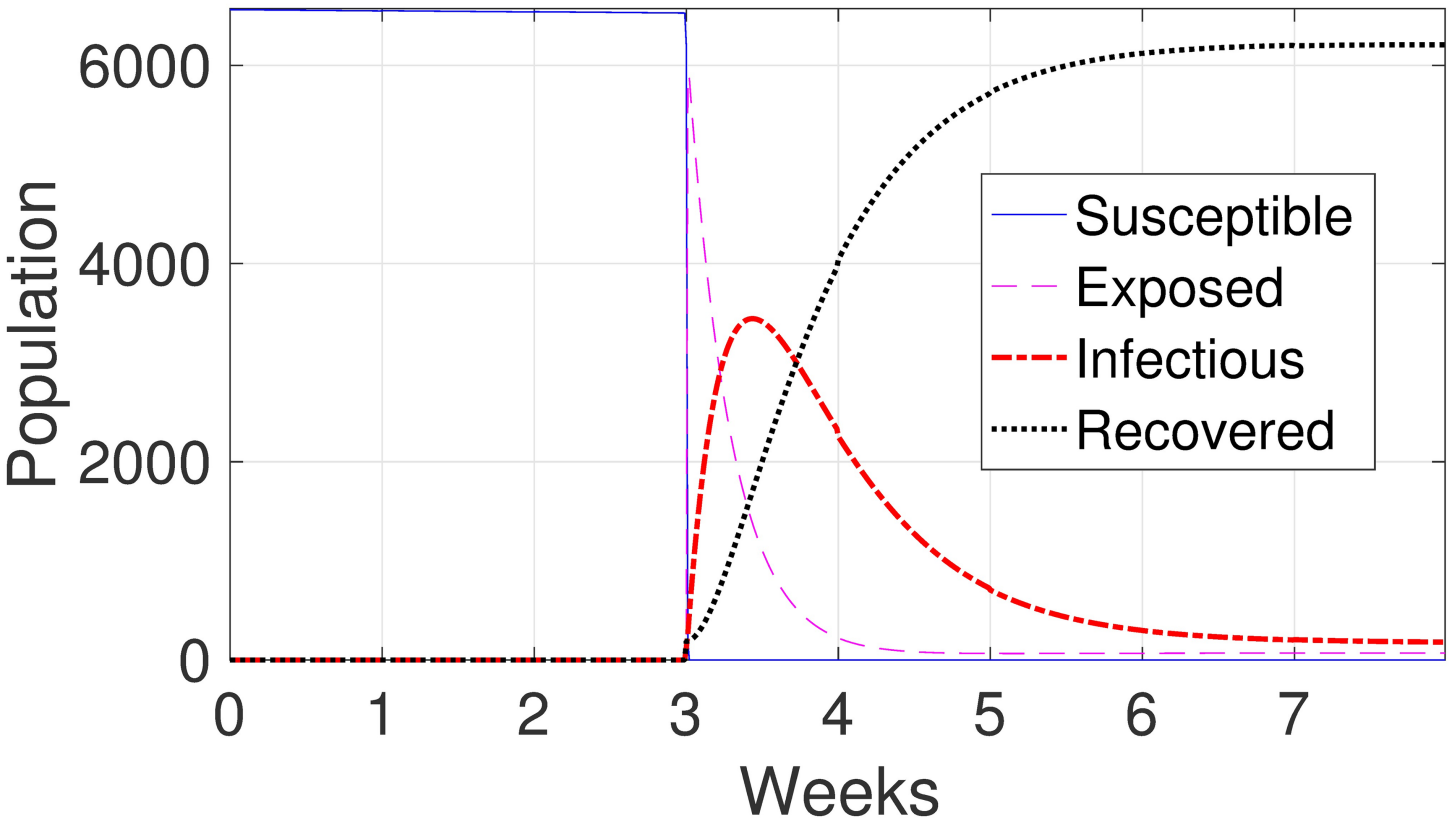
Model results of assuming *β*_*ind*_ = 0 for growing pigs. The panel shows a delay in the infection peak of about 25 days and no change in the number of infectious animals compared to the case of without reduction of indirect contact as in Fig 2d.

To test reduction of indirect contact, we set *β*_*ind*_ to zero in the model. The results show the delay of the spread of the disease in the farm. There is no infection in the growing pigs until 25 days when the infection increases sharply to a spike (see Fig 7).

### Sensitivity analysis

**Fig 8:**
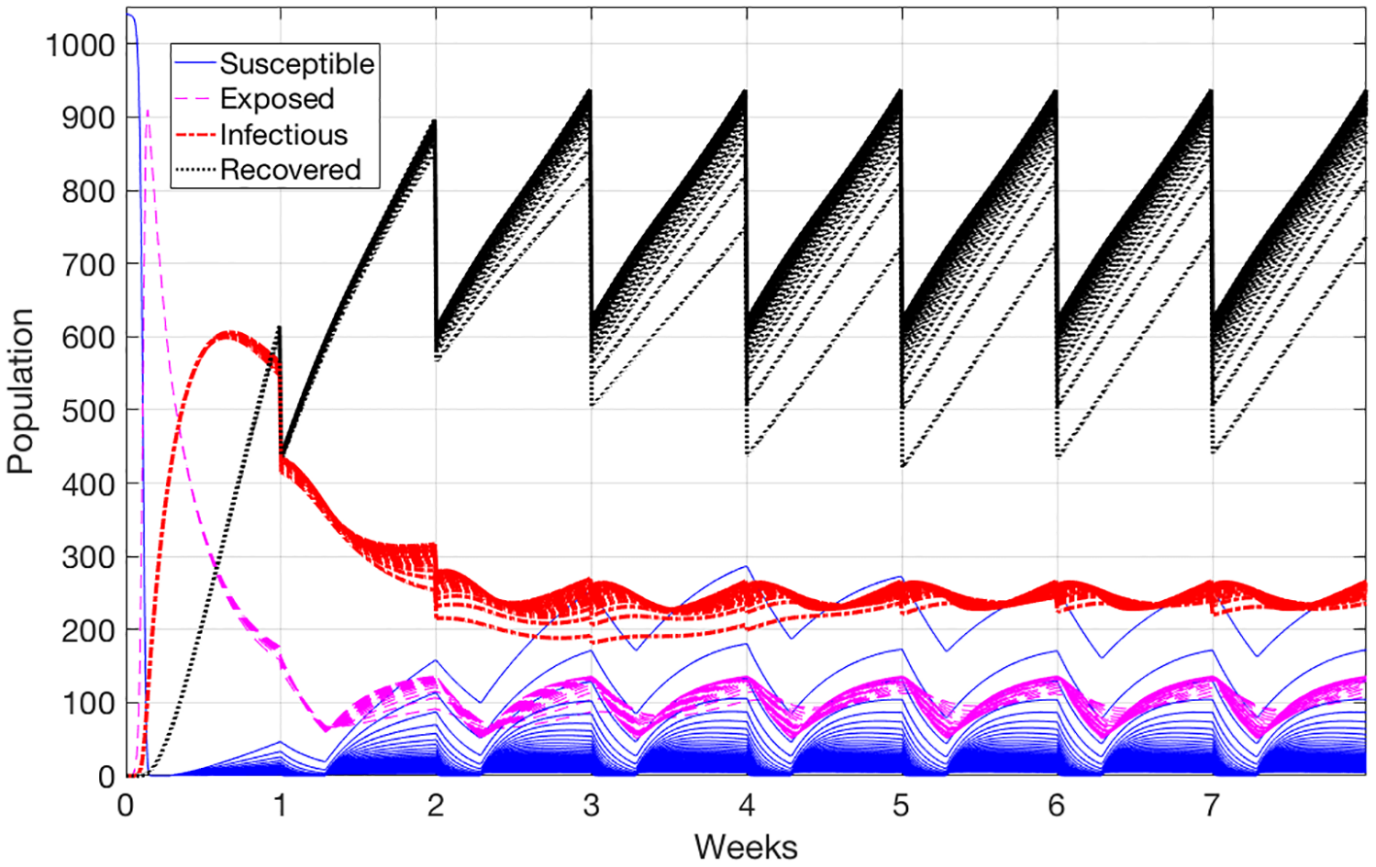
Model results by uniformly varying the range of direct transmission rate for piglets with immunity parameter.

Varying all the control parameters, i.e., the direct and indirect transmission rates for pigs, has no significant effect on the model behavior, and the number of infectious animals associated with all of these parameters is almost identical. However, we notice that when we change the direct transmission rate for piglets with immunity $\beta_{d}^{pm}$, many more piglets are susceptible. Fig 8 shows the results of 100 uniformly sampled values in the range of (0.001 − 0.061). This larger number of susceptible animals is only notice when $\beta_{d}^{pm}$ is very small. As a result of the facts that the virus transmission is small, and the piglets are already immune, a larger portion of the newborn piglets stays in the susceptible state for a longer time.

## Discussion

Several studies reported high prevalence of the IAV in pigs in different regions of the world, such as Europe [6] East Asia [7] and North America [8, 27]. Specifically, it is observed by Poljak et al. [8, 22] that pigs in Ontario are positive to IAV virus and the prevalence is increasing over time. Our study illustrates the transmission of IAV and the reinfection within a farrow-to-finish swine farm in Ontario. A mathematical SEIR model presented in [12] is extended and implemented. Simulation results indicate that in a fully populated farm an IAV outbreak through the farm causes the persistence of the infection within the piglet and gilt populations. The disease was observed at a high level in the piglets even though they had maternal immunity from immune sows. The virus persisted in the piglet population due to the continuous supply of new piglets being born each week, while in the gilt population was due to weekly incoming susceptible gilts. Moreover, as a result of the incoming recovered individuals each week, the disease died out among the sows and growing pigs.

Our finding is in agreement with other experimental studies where the major IAV infection takes place in the piglets [7, 28]. Furthermore, the same observation was also observed at a breeding farm in the modelling studies performed by Reynolds et al. [12] and White et al. [21]. We conclude that the persistence of the virus in the farrow-to-finish farm is due to the supply of new susceptible pigs. This observation has been also reported by [19], however, we are utilizing different methods and assumptions. Moreover, our results agree with the empirical results of [29], in which they also find out that the progress of the influenza outbreak through the farm is within three weeks.

Furthermore, we also studied the reinfection scenario, in which the recovered pigs could be susceptible to receiving the virus once more. We studied the effect of *ω* by changing its value from 1/50 to 1/200. We noticed that the number of infectious pigs decreases as this value decreases, but the virus is still in circulation even though with the duration of immunity is long (*ω* = 1/200). Results revealed that the disease was endemic in the entire farm, and unlike the typical infection scenario, the virus persisted among the growing pigs and sows.

The fact that widespread IAV infection was confirmed and the disease was maintained in the farm, raises the question of what are the efficient strategies to control the spread of the disease. Vaccination is the most common strategy that is used to minimize the transmission of a disease. Another strategy is the reduction of contact within the population. Several studies showed that vaccination can reduce the transmission of IAV virus but it does not completely element it [2, 21, 30].

Clinically, veterinarians are using vaccination of gilts to help control influenza circulation in a herd. Although this could result in abortion, it is relatively a common practice. The second vaccination strategy is to vaccinate breeding sows before lactation. With this strategy, farmers are trying to maximize maternal immunity of newly born piglets, and it is done continuously in a herd. Another vaccination strategy is mass vaccination, where all sows, or all sows and piglets, are vaccinated at one time. It is not done frequently, but the goal is to eliminate infection from a herd by creating a high level of immunity in all animals. This strategy is only applied in breeding herds. The reducing contact strategy, which is called McRebel strategy, is applied to any infectious disease of pigs. The aim of this strategy is to reduce contact between animals and prevent infection. We refer the reader to [31, 32] for further details about these strategies.

In this study, we modify the dynamics of the model to apply these strategies. We investigate the effect of these strategies, and particularly on a farrow-to finish herd, which is unique and challenging because animals of different ages are at the same location. It is easy to eliminate infection from farms where animals are segregated by age, but in farrow-to-finish facilities, this is a real challenge. The decision whether or not to apply these strategies will be made depending on their cost and their effectiveness, which is the reason for this study.

Based on our model, these vaccination strategies are incapable of reducing the influenza infection on the whole farm. Especially among the piglets, the infection level remains high, as they become infected almost immediately after they are born. In the continuous case, when the vaccination is applied every week, and since naturally all incoming gilts are susceptible, vaccinating of these gilts is effective in the reduction of the infectious gilts. In contrast, the pre-farrow vaccination does not show any change in the number of infectious in the farm. This is because there is almost no susceptible sows to be vaccinated, as most of them are already in the recovered group. Mass vaccination (single discrete case) reduce only the infectious level in the gilts for one week, then it returns back to endemic equilibrium. This is expected since new gilts are entering the farm every week and vaccination has not been applied to the new comers. Furthermore, mass vaccination has no effect to the rest of the pigs, since at any given time most of the animals are already in recovered state. In practice the vaccination may be more effective than the observed effects in our model. This is because the information about how long the pigs have been in the recovered state are not captured in this model, so every pig has an equal probability of moving from the recovered group to the susceptible group.

Reynolds et al. [12] also suggested using vaccination strategies to reduce the influenza transmission in a breeding and wean-to-finish farm. They found that these strategies are ineffective in reducing the virus in the breeding herd, but caused a small reduction in infectious pigs in the wean-to-finish farm. They modeled these strategies by using the transmission parameter *β*. In our study, this scenario is evaluated by moving susceptible animals to a recovered state where reinfection can occur. In the lack of empirical data about the immunity time of the vaccine, we assume that the immunity time for the vaccine is the same as the natural immunity which is 180 days.

Likewise, regarding the strategy of reduction in indirect contact, our model indicates that this strategy is also ineffective in reducing the level of infection in the farm. It resulted in only the delay of the spread of the disease in the farm. The disease does not die out due to the weekly continuous movement of the pigs through the farm. It is also observed that there is no delay or change among the gilts (see Fig 2a) since the disease starts in the gilts room.

Sensitivity analysis is implemented to test the effect of variation the direct and indirect transmission rates in the farm. Despite the variation of these parameters the IAV is still persistence in the farm in particular between piglets population.

A major limitation of this study is that there is no empirical data for many parameters that affect the behavior of the model such as the vaccine immunity time and the indirect transmission rates. However, the same exact model can be applied once such data becomes available. Another limitation of this study is that, for simplicity, we only focus on a single influenza strain and we are not aware of any modeling study of pig farms considering multiple influenza strains.

In conclusion, the dynamics of IAV virus is not fully understood and the disease is maintained in the farm specifically in the piglet population, which is a serious concern for public health. The effectiveness of vaccination strategies is still questionable. Reducing the indirect contact results in delaying the disease, however, it is also not able to reduce the virus to an acceptable level. A high level of infection in the animals could cause high risks to humans and other species. Therefore, public awareness about this virus should be increased. This requires better understanding of how other factors, such as farm management practices and the interaction of the farm workers with the pigs, can contribute to the persistence of the disease in the swine. We argue that, by fully understanding the dynamics of the IAV virus, most of the limitations can be successfully addressed and resolved. Therefore, more comprehensive experimental studies are required to cover this gap.
